# Translating Exosomal microRNAs from Bench to Bedside in Parkinson’s Disease

**DOI:** 10.3390/brainsci15070756

**Published:** 2025-07-16

**Authors:** Oscar Arias-Carrión, María Paulina Reyes-Mata, Joaquín Zúñiga, Daniel Ortuño-Sahagún

**Affiliations:** 1 División de Neurociencias|Clínica, Instituto Nacional de Rehabilitación Luis Guillermo Ibarra Ibarra, Mexico City 14389, Mexico; 2 Tecnologico de Monterrey, Escuela de Medicina y Ciencias de la Salud, Mexico City 14380, Mexico; joaquin.zuniga@iner.gob.mx; 3 Departamento de Disciplinas Filosófico, Metodológicas e Instrumentales, CUCS, Universidad de Guadalajara, Guadalajara 44350, Mexico; paulina.reyes@academicos.udg.com; 4 Laboratorio de Inmunobiología y Genética, Instituto Nacional de Enfermedades Respiratorias Ismael Cosío Villegas (INER), Mexico City 14080, Mexico; 5 Laboratorio de Neuroinmunobiología Molecular, Instituto de Neurociencias Translacionales, CUCS, Universidad de Guadalajara, Guadalajara 44350, Mexico; daniel.ortuno@academicos.udg.mx

**Keywords:** Parkinson’s disease, exosomes, microRNAs, oxidative stress, neuroinflammatory response, biomarkers, gene therapy, extracellular vesicles

## Abstract

Parkinson’s disease (PD) is a progressive neurodegenerative disorder marked by dopaminergic neuronal loss, α-synuclein aggregation, and chronic neuroinflammation. Recent evidence suggests that exosomal microRNAs (miRNAs)—small, non-coding RNAs encapsulated in extracellular vesicles—are key regulators of PD pathophysiology and promising candidates for biomarker development and therapeutic intervention. Exosomes facilitate intercellular communication, cross the blood–brain barrier, and protect miRNAs from degradation, rendering them suitable for non-invasive diagnostics and targeted delivery. Specific exosomal miRNAs modulate neuroinflammatory cascades, oxidative stress, and synaptic dysfunction, and their altered expression in cerebrospinal fluid and plasma correlates with disease onset, severity, and progression. Despite their translational promise, challenges persist, including methodological variability in exosome isolation, miRNA profiling, and delivery strategies. This review integrates findings from preclinical models, patient-derived samples, and systems biology to delineate the functional impact of exosomal miRNAs in PD. We propose mechanistic hypotheses linking miRNA dysregulation to molecular pathogenesis and present an interactome model highlighting therapeutic nodes. Advancing exosomal miRNA research may transform the clinical management of PD by enabling earlier diagnosis, molecular stratification, and the development of disease-modifying therapies.

## 1. Introduction

Parkinson’s disease (PD) is the second most prevalent neurodegenerative disorder, affecting approximately 1% of individuals over the age of 60 and up to 4% of those over 85 years [1]. It is primarily characterized by the progressive loss of dopaminergic neurons in the substantia nigra pars compacta and by the pathological accumulation of α-synuclein aggregates. These changes lead to hallmark motor symptoms, including bradykinesia, resting tremor, rigidity, and postural instability [2,3]. However, PD is now widely recognized as a multisystem disorder, with non-motor symptoms—including cognitive impairment, mood disturbances, autonomic dysfunction, and sleep disorders—often preceding the onset of motor deficits and contributing substantially to disease burden [4,5,6]. Despite advances in the understanding of PD pathophysiology, available treatments remain symptomatic and fail to halt disease progression or prevent neuronal loss [7].

Recent studies have identified microRNAs (miRNAs)—small, non-coding RNA molecules that regulate gene expression post-transcriptionally—as potential biomarkers and therapeutic targets in PD. These molecules are key modulators of several pathophysiological mechanisms implicated in neurodegeneration, including mitochondrial dysfunction, oxidative stress, neuroinflammation, and apoptosis [8]. Aberrant miRNA expression profiles have been observed in human biofluids and post-mortem brain tissue, while experimental models confirm their role in neuroprotection and the regulation of inflammation. For instance, miR-7 inhibits α-synuclein aggregation [9], miR-124 suppresses microglial activation [10], and miR-29c-3p attenuates neuroinflammation by targeting the NLRP3 inflammasome [11]. Conversely, pro-pathogenic miRNAs such as miR-34a and let-7a exacerbate oxidative damage and dopaminergic cell loss, with the latter promoting neurotoxicity through Toll-like receptor (TLR) activation [12].

The discovery of exosomal miRNAs—miRNAs encapsulated within extracellular vesicles—has further expanded our understanding of PD pathophysiology and biomarker development. Exosomes mediate intercellular communication and are capable of crossing the blood–brain barrier (BBB), positioning them as attractive candidates for non-invasive diagnostic tools and therapeutic vehicles [13]. Several cerebrospinal fluid (CSF) and blood-derived exosomal miRNAs, including miR-331-5p and miR-505, have demonstrated high diagnostic accuracy in distinguishing PD from other neurodegenerative diseases (area under the curve [AUC] > 0.85) [14]. Additional candidates, such as miR-19b, miR-24, and miR-195, can differentiate PD from vascular parkinsonism with high specificity [12].

Beyond diagnostics, exosomal miRNAs hold therapeutic potential. In animal models of PD, exogenous delivery of neuroprotective miRNAs has been shown to ameliorate disease pathology. For example, miR-7116-5p reduces TNF-α overproduction and preserves dopaminergic neurons [15], while miR-29c-3p prevents microglia-induced neurotoxicity via NLRP3 inhibition [11]. These findings suggest that exosome-based miRNA delivery systems may represent a novel class of disease-modifying therapies.

Nonetheless, several challenges remain in translating exosomal miRNA research into clinical applications. Major obstacles include the lack of standardized protocols for exosome isolation, limited reproducibility of miRNA quantification techniques, and incomplete understanding of miRNA cargo selection and release mechanisms [13]. Additionally, ensuring the stability, bioavailability, and selective uptake of exosomal miRNAs by target cells—especially degenerating dopaminergic neurons—remains a key limitation for therapeutic development [14].

Advances in bioengineering, nanotechnology, and RNA therapeutics may help address these barriers by enabling precise delivery, enhancing therapeutic stability, and minimizing immunogenicity and off-target effects. This review synthesizes recent progress in the application of exosomal miRNAs as diagnostic and therapeutic tools in PD, with a particular focus on their potential for disease modification. We discuss current limitations, outline technological solutions, and propose strategic directions to accelerate the clinical translation of exosomal miRNA-based approaches. Bridging fundamental research with translational innovation may, ultimately, enable the integration of miRNA-based strategies into precision medicine frameworks for PD and related neurodegenerative diseases.

## 2. Data Sources

To ensure a comprehensive and up-to-date synthesis, we conducted a structured literature search across the PubMed, Web of Science, and Scopus databases, focusing on peer-reviewed articles published in English between January 2020 and May 2024. Earlier seminal publications were selectively included when foundational to mechanistic or conceptual frameworks.

The search strategy combined MeSH terms and free-text keywords using Boolean operators, with the following terms: “exosomal microRNAs”, “Parkinson’s disease”, “dopaminergic neurodegeneration”, “biomarkers”, “oxidative stress”, “neuroinflammation”, “α-synuclein”, “mitochondrial dysfunction”, “extracellular vesicles”, “synaptic dysfunction”, and “gene regulation”. Two independent reviewers screened search results.

We included studies that met the following inclusion criteria: (i) original research articles or systematic reviews; (ii) use of validated exosome isolation techniques; and (iii) focus on exosomal miRNAs in the context of PD as biomarkers, mechanistic modulators, or therapeutic targets. Exclusion criteria included the following: (i) studies exclusively examining intracellular, free-circulating, or leukocyte-derived miRNAs; (ii) lack of experimental or clinical relevance to PD; and (iii) incomplete methodological information.

To increase clarity, we explicitly distinguished between exosomal miRNAs and other classes of circulating miRNAs throughout the review. Where relevant, findings based on non-exosomal miRNAs were included only to contextualize mechanistic pathways or identify research gaps, and these were clearly labeled as such.

We critically evaluated the methodological rigor of included studies, particularly in terms of exosome isolation (e.g., ultracentrifugation, SEC, and immunoaffinity), miRNA profiling (e.g., qPCR, NGS, and ddPCR), and biomarker validation. Both preclinical models and clinical datasets were examined to capture the translational spectrum, including studies evaluating diagnostic accuracy (e.g., ROC curves and AUC values) and longitudinal progression markers.

This integrative and methodologically rigorous approach ensures that the review reflects the current landscape of exosomal miRNA research in PD, identifies areas of consensus and controversy, and provides a foundation for future translational efforts in diagnostics and therapeutics.

## 3. Advanced Exosome Isolation Techniques and Characterization of miRNA Expression

**Figure 1:**
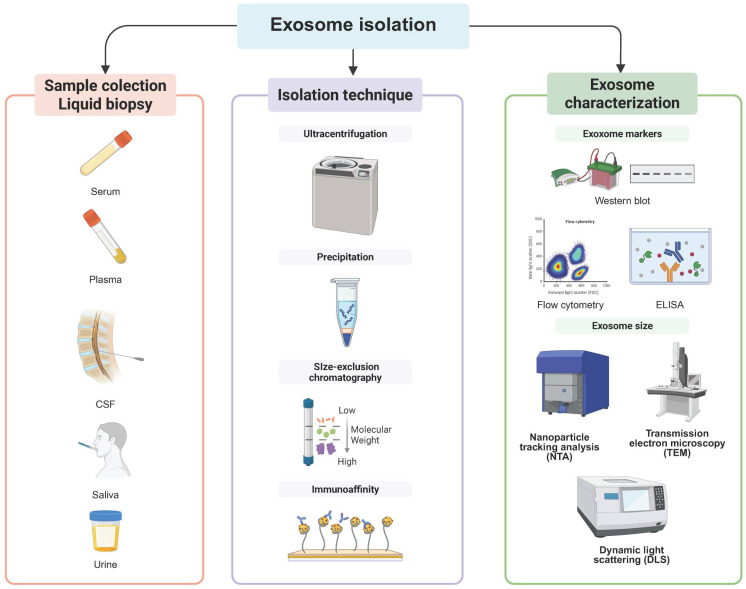
Workflow for the isolation and analysis of exosomal microRNAs in Parkinson’s disease. Stages of exosome isolation used in Parkinson’s disease research. **Left** panel: biofluid collection for liquid biopsy, including serum, plasma, cerebrospinal fluid (CSF), saliva, and urine. **Middle** panel: isolation techniques to enrich for exosomes, including ultracentrifugation, precipitation, size-exclusion chromatography, and immunoaffinity capture. **Right** panel: characterization of isolated exosomes using Western blot and ELISA to confirm exosomal markers, flow cytometry for vesicle surface profiling, and size determination through nanoparticle tracking analysis (NTA), transmission electron microscopy (TEM), and dynamic light scattering (DLS). This workflow enables robust isolation and characterization of exosomes for downstream miRNA profiling. Created in BioRender.com.

Exosomal miRNAs have emerged as promising biomarkers and therapeutic candidates in PD; however, their clinical application is hindered by major technical challenges related to exosome isolation and accurate miRNA expression profiling. Exosomes are heterogeneous, nanoscale vesicles that are often purified together with other extracellular vesicles and contaminants, requiring highly specific isolation methods [16] (Figure 1). Ultracentrifugation is still the most commonly used approach. It utilizes high-speed centrifugation and density gradients to separate exosomes based on their size and density. However, this method is limited by the potential for damage to the vesicles and loss of miRNA content due to shear forces [17]. It also isolates non-exosomal vesicles.

Alternative strategies have been developed to improve the purity and integrity of exosomes. Size-exclusion chromatography (SEC), in which vesicles are separated based on their size, enables high recovery rates while preserving the exosomal structure and RNA cargo, making it particularly advantageous for miRNA analysis [18]. Immunoaffinity separation with antibodies targeting exosomal surface markers, such as CD63, CD81, and CD9, offers higher specificity and allows for the selective isolation of vesicles of different cellular origin [19,20]. However, it is labor-intensive and costly. Then again, no single isolation technique guarantees absolute purity, so a multi-step, integrative, and complementary approach tailored to specific research or clinical applications is often required (Table 1).

**Figure 2:**
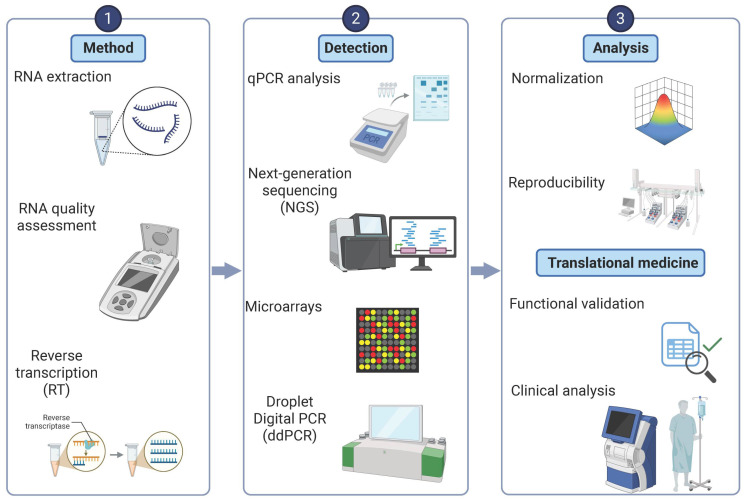
Workflow for microRNA detection and analysis in Parkinson’s disease. An integrated pipeline for exosomal microRNA analysis. **Left** panel: RNA extraction from isolated exosomes and assessment of RNA quality. **Middle** panel: detection approaches including quantitative PCR (qPCR), next-generation sequencing (NGS), microarrays, and droplet digital PCR (ddPCR), enabling both targeted and high-throughput miRNA profiling. **Right** panel: analysis steps, including normalization of miRNA expression data, assessment of reproducibility across experiments, and functional validation in biological systems. Translational applications include the use of exosomal miRNAs as biomarkers for disease diagnosis, monitoring progression, and evaluating treatment response in Parkinson’s disease. Created in BioRender.com.

Once exosomes are isolated, accurate miRNA quantification and characterization are critical for biomarker validation and therapeutic research (Figure 2). Commercial RNA extraction kits, such as the miRNeasy Micro Kit, have improved the recovery of small RNAs while minimizing degradation. Quantitative polymerase chain reaction (qPCR) is the most widely used technique due to its high sensitivity for detecting specific miRNAs, making it particularly valuable for validating clinical biomarkers. However, its dependence on pre-selected targets limits its ability to capture the full complexity of the miRNA landscape in PD [19] (Table 2).

**Table 1:** Key criteria for exosome isolation methods applicable in Parkinson’s disease research.

Criteria	Method	Details	Key Considerations
Sample collection		Blood (5–10 mL) or CSF (1–2 mL) in sterile tubes	Use EDTA-coated tubes for plasma to prevent clotting; process promptly or store at −80 °C to preserve RNA integrity.
	Pre-clearing	Centrifugation (3000× *g*, 15 min, 4 °C) → filtration (0.22 μm)	Additional spin at 12,000× *g* for plasma removes large extracellular vesicles and debris.
Isolation technique			
	Ultracentrifugation	10,000× *g* (30 min) → 100,000× *g* (70 min, 4 °C)	Balance tubes carefully; filter PBS (0.22 μm) before washing. Verify speed, time, and temperature.
	Precipitation		May co-precipitate non-exosomal material. Validate purity (e.g., via Western blot).
	Size-exclusion chromatography (SEC)	Load sample onto Sepharose CL-2B column → collect 500 µL fractions	Analyze fractions using nanoparticle tracking analysis (NTA) or dynamic light scattering (DLS) techniques.
	Immunoaffinity capture	Incubate with magnetic beads (CD63/CD81/CD9) for 2–4 h at 4 °C	Select antibodies based on exosomal origin (neuronal and microglial); gentle washes prevent bead loss.
Exosome characterization			
	Western blot, ELISA, or flow cytometry	Confirm exosomal markers (e.g., CD63, CD81, and TSG101)	Negative markers (e.g., calnexin) should be absent to rule out contaminants.
	Nanoparticle tracking analysis (NTA), transmission electron microscopy (TEM), or dynamic light scattering (DLS)	Confirm exosome size (30–150 nm) and morphology	
	Yield quantification	Protein concentration or particle number	

Key Takeaways:

1. Depending on the source of the biofluid (e.g., plasma, serum, and cerebrospinal fluid), the specific conditions for preservation and isolation may vary.
2. Sample storage is critical for exosome integrity. Proper handling of CSF and plasma samples (e.g., using EDTA or citrate, storage at −80 °C, and limiting freeze–thaw cycles) minimizes RNA degradation and preserves miRNA content for analysis.
3. Methods for isolating exosomes vary in terms of purity, yield, and specificity, which affect subsequent miRNA analysis. High-quality studies should provide detailed protocols and validate the purity of exosomes.
4. Isolation methods affect the purity of exosomes. Ultracentrifugation, SEC, and immunoaffinity capture each have their strengths and limitations and require an integrated approach for optimal exosome recovery in PD research.
5. Studies should adhere to the MISEV2018 guidelines (e.g., when specifying isolation methods, EV characterization and terminology such as “exosomes” vs. “EVs”)

**Table 2:** miRNA expression detection methods workflow that can be used in Parkinson’s disease research.

Method	Details	Key Considerations
RNA extraction	Lyse exosomes with an exosome-specific RNA isolation kit (e.g., miRNeasy Micro Kit).	Include spike-in controls for normalization; follow low RNA input protocols to avoid contamination from free-circulating miRNAs.
RNA Quality Assessment	Quantify RNA using Qubit fluorometer RNA Assay → assess integrity with a bioanalyzer.	Unlike total RNA, exosomal RNA lacks rRNA peaks; check A260/280 ratios for purity.
Reverse transcription (RT)	Use specific Kits (e.g., miRCURY LNA RT Kit and miScript II RT Kit).	Use technical replicates and non-template controls to minimize variability and normalize extraction efficiency (e.g., spike-in RNAs, such as cel-miR-39).
Detection		
qPCR analysis	Most common for targeted miRNA analysis.	Specify primers, cycling conditions, and reference genes (e.g., U6 and miR-16)
Next-generation sequencing (NGS)	Construct and evaluate small RNA libraries (e.g., Illumina TruSeq and Ion Total RNA-Seq), sequencing depth, and data preprocessing (e.g., adapter trimming and quality filtering).	Remove adapter dimers before sequencing and validate the findings via qPCR. Bioinformatics analysis is fundamental.
Microarrays	Less common but used for high-throughput screening. Hybridize miRNA samples to the probe chip.	Ensure high RNA quality to avoid non-specific binding.
Droplet Digital PCR (ddPCR)	Emerging for absolute quantification.	Check optimization details (e.g., primer concentrations and annealing temperatures).
Normalization	Use endogenous (e.g., U6 and miR-16) or exogenous (e.g., cel-miR-39) reference genes.	Validate differential expression (e.g., via independent qRT-PCR or cross-platform comparison). Clearly describe statistical methods for the analysis of the differential expression (e.g., edgeR and t-tests).
Reproducibility	Report Technical and biological replicates.	Annotate raw data availability (e.g., GEO database deposition).
Functional validation	Transfect dopaminergic neurons (SH-SY5Y and primary cultures) with miRNA mimics/inhibitors.	Assess oxidative stress and neuroinflammation, as well as confirm targets using quantitative PCR (qPCR) and Western blot.
Clinical analysis	Quantify exosomal miRNAs in patient plasma or CSF.	Correlate miRNA levels with PD progression and clinical parameters.

Key Takeaways:

1. RNA extraction must be optimized for samples with low yields. Exosomal RNA has a low concentration and lacks rRNA peaks, so specialized RNA purification kits and quality control steps are required.
2. qPCR is effective for biomarker validation, while NGS enables the discovery of new biomarkers. qPCR is highly sensitive for known miRNAs, while NGS enables unbiased profiling that allows for identification of novel miRNA signatures in PD.

Next-generation sequencing (NGS) offers an unbiased, high-throughput approach that facilitates the identification of novel, low-abundance microRNAs (miRNAs) involved in the pathogenesis of PD [20]. While microarray platforms offer a cost-effective alternative for large-scale screening, they lack the resolution of NGS and are prone to cross-hybridization artefacts. The variability of RNA extraction protocols, sample preparation, and data normalization further complicates a comparison between different studies and underscores the urgent need for standardized methods [21]

New technologies, such as high-throughput miRNA assays and CRISPR–Cas9 screening, have begun to map miRNA regulatory networks, providing insights into their molecular targets and disease relevance [22]. Standardizing methods for isolating exosomes and profiling miRNA remains a top priority to ensure the reproducibility and clinical applicability of miRNA-based exosomal diagnostics and therapeutics in PD [16].

## 4. miRNAs in Animal Models of Parkinson’s Disease

Emerging evidence from preclinical models highlights the role of miRNAs in modulating key pathogenic mechanisms in PD, including neuroinflammation, oxidative stress, and survival of dopaminergic neurons [9,11,14,15,23,24,25,26] (Table 3). These small, non-coding RNA molecules exert different effects, with some exhibiting neuroprotective properties, while others contribute to disease progression by exacerbating inflammatory and degenerative processes.

Among miRNAs with neuroprotective potential, miR-7, miR-30e, and miR-124 attenuated neuroinflammation and preserved dopaminergic neurons in MPTP-induced PD models [23,26,27]. Both miR-7 and miR-30e downregulate the NLRP3 inflammasome, a key mediator of neuroinflammatory responses. In contrast, miR-124 inhibits microglial activation via the MEKK3/NF-κB signaling pathway, thereby reducing the inflammatory burden. Additionally, miR-190 mitigates oxidative stress and inflammatory signaling, resulting in neuroprotection and delaying neurodegeneration [25].

Conversely, other miRNAs appear to exacerbate the pathological processes underlying PD. miR-195, which is overexpressed in activated microglia, promotes ROCK1 signaling, a pathway associated with neuroinflammation and neuronal damage [28]. Similarly, miR-29c-3p and miR-155-5p regulate inflammatory cascades, including the NLRP3 inflammasome and the SOCS1/Nrf2 signaling pathway, altering microglial reactivity and immune responses [11,24]. In contrast, let-7a, which is generally associated with immune regulation, inhibits neuroinflammation triggered by α-synuclein. This suggests that the effects of miRNAs may be context-dependent and influenced by the disease stage or cellular environment [9].

These findings emphasize the multisystemic nature of PD and highlight miRNAs as potential biomarkers and therapeutic targets. Given their role in regulating inflammation and neuron survival, miRNA-based interventions may represent a disease-modifying strategy that goes beyond symptom relief. However, further studies are needed to establish their therapeutic efficacy, optimize delivery methods, and determine their long-term impact in a clinical setting.

**Table 3:** MicroRNAs modulating pathways in animal models of Parkinson’s disease.

miRNA	Experimental Model and Target Pathway	Expression in PD	Key Findings	Translational Implications and Reference
miR-7116-5p	MPP^+^-treated microglia; TNF-α	↓ Downregulated	Sensitizes TNF-α production, driving neuronal damage	Target to reduce TNF-α neurotoxicity [15]
miR-330	Microglia differentiation; SHIP1/NF-κB	↓ Downregulated	Regulates microglial polarization and inflammation	Target for microglial modulation [29]
miR-195	LPS-stimulated BV2 cells; ROCK1	↑ Upregulated	Triggers neuroinflammation via ROCK1	ROCK1 inhibition may attenuate inflammation [28]
miR-29a	MPP^+^-treated SH-SY5Y; GSK-3β/MAVS	↑ Upregulated	Neuroprotective: reduces ROS, α-synuclein, and cell death	Potential therapeutic target [30,31]
miR-29c-3p	LPS-BV2/MPP^+^-SH-SY5Y/MPTP; NFAT5/NLRP3/TET2	↓ Downregulated	Suppresses microglial activation and regulates autophagy	NFAT5/NLRP3, autophagy regulation for PD therapy [11,32]
miR-29c	MPP^+^-SH-SY5Y/MPTP; Sp1	↓ Downregulated	Protects against neuroinflammation and apoptosis	Potential PD biomarker and therapeutic target [11]
miR-124	MPP^+^-SH-SY5Y/MN9D/MPTP; Bim/Calpain1-cdk5/FSTL1	Mixed (↑/↓)	Protects dopaminergic neurons; modulates oxidative stress, inflammation, and apoptosis	Promising therapeutic target [33,34]
miR-20a-5p	LPS-BV2/MPTP; STAT3, α-synuclein	↑ Upregulated	Alleviates neuronal damage and inflammation	Modulates cell viability and inflammatory responses [35]
miR-93	LPS-BV2/MPTP; STAT3	↑ Upregulated	Reduces apoptosis, microglial activation, and inflammation	Potential for protecting substantia nigra neurons [32]
miR-218-5p	MPP^+^-BV2/MPTP/6-OHDA; Ddx41/IFN-I/LASP1	↑/↓ Mixed	Alleviates microglial inflammation, dopaminergic neuron loss, and motor dysfunction	Target to improve DA neuron survival [36,37]
miR-221-3p	MPP^+^-MN9D/MPTP; NPTX2	↑ Upregulated	Modulates autophagy by reducing NPTX2	Potential PD biomarker [38]
miR-101a-3p	MPP^+^-Neuro-2a/MPTP; ROCK2	↑ Upregulated	Improves neurological function; reduces α-synuclein accumulation	Therapeutic potential [39]
miR-335	LPS-BV2/MPTP; LRRK2	↓ Downregulated	Attenuates chronic neuroinflammation	May slow PD progression [40]
miR-7	MPTP; NLRP3	↑ Upregulated	Suppresses neuroinflammation	Anti-inflammatory strategy in PD [26]
miR-30e	MPTP; NLRP3	↑ Upregulated	Reduces neuroinflammation and neuronal damage	Neuroprotective intervention [23]
miR-124	MPTP; p62/p38/MEKK3/NF-κB	↓/↑ Mixed	Inhibits neuroinflammation and modulates autophagy	Potential anti-inflammatory therapy [27,41]
miR-150	PD patients and LPS microglia; AKT	↓ Downregulated	Modulates neuroinflammation; potential biomarker	Early diagnostic candidate [42]
miR-190	MPTP; NLRP3	↑ Upregulated	Protects against neuronal damage and inflammation	Antioxidant and anti-inflammatory potential [25]
let-7a	α-Synuclein overexpression; STAT3	↓ Downregulated	Inhibits α-synuclein-induced inflammation	Candidate for mitigating α-syn neuroinflammation [9]
miR-185	6-OHDA; IGF1	↓ Downregulated	Ameliorates DA neuron damage via PI3K/AKT	Enhances IGF1-mediated neuroprotection [43]
miR-375	6-OHDA; Sp1	↑ Upregulated	Ameliorates dopaminergic neuron damage; reduces oxidative stress	SP1 as a biomarker and therapeutic target [44]
miR-155-5p	MPTP; SOCS1/Nrf2	↑ Upregulated	Alleviates inflammation, apoptosis and oxidative stress	Targeted by rosmarinic acid for neuroprotection [24]

Key Takeaways:

1. The NF-κB pathway acts as a central hub, with molecules such as MEKK3, SHIP1, SOCS1, GSK3B, TNF-α, MAVS, and NLRP3 directly or indirectly modulating its activity.
2. The JAK/STAT pathway also connects STAT3, SOCS1, and PDK1/IFN, while AKT and TET2/autophagy provide regulatory feedback.
3. FoxO1 could provide regulatory feedback by interacting with both pathways. Its role is particularly notable in anti-inflammatory signaling via SOCS1 upregulation and autophagy induction, which aligns with TET2’s effects.
4. AKT and autophagy (via TET2 and FoxO1) provide additional regulatory layers to balance proinflammatory and anti-inflammatory responses.
5. These pathways collectively balance proinflammatory and anti-inflammatory responses.

## 5. Circulating and Exosomal miRNAs in Parkinson’s Disease

Exosomal miRNAs have gained attention as promising biomarkers for PD, with potential implications for diagnosis and monitoring [12,13,27,45]. These miRNAs, detectable in CSF, plasma, and serum, exhibit disease-specific expression patterns that distinguish PD from healthy controls and other neurodegenerative diseases (Table 4).

In CSF-derived exosomes, levels of miR-1 and miR-19b-3p are significantly reduced in patients with PD, while miR-153, miR-409-3p, and miR-10a-5p are elevated [13]. These miRNAs regulate the integrity of dopaminergic synapses and neurotrophic signaling, which are central to the pathophysiology of PD. Remarkably, miR-409-3p has the highest diagnostic accuracy (AUC = 0.970), followed by let-7g-3p (AUC = 0.950) and miR-10a-5p (AUC = 0.900), indicating its potential as a non-invasive biomarker.

Similarly, plasma-derived exosomal miRNAs provide additional discriminatory power. miR-331-5p is upregulated in PD (AUC = 0.849), while miR-505 is downregulated (AUC = 0.898), suggesting that these miRNAs are involved in synaptic dysfunction and neurodegeneration [27]. Altered expression patterns are also observed for exosomal miRNAs in serum, with miR-195, miR-19b, and miR-24 showing disease-associated changes. Among them, miR-24 has the highest diagnostic accuracy (AUC = 0.908) [12].

**Table 4:** Circulating and exosomal microRNAs in Parkinson’s disease.

miRNA	Sample Type and Study Population	Expression in PD	Key Findings	Diagnostic Potential and Reference
miR-1	CSF exosomes (47 PD, 28 AD, 27 controls)	↓ Downregulated	Decrease in PD; involved in neurotrophin signaling	High specificity for PD diagnosis [13]
miR-19b-3p	CSF exosomes (47 PD, 28 AD, 27 controls)	↓ Downregulated	Decrease in PD; part of the dopaminergic synapse pathway	AUC = 0.705; sensitivity 94%, specificity 95% [13]
miR-153	CSF exosomes (47 PD, 28 AD, 27 controls)	↑ Upregulated	Increased in PD; linked to dopaminergic synapse regulation	AUC = 0.780; high sensitivity [13]
miR-409-3p	CSF exosomes (47 PD, 28 AD, 27 controls)	↑ Upregulated	Increased in PD; involved in neurotrophin pathways	AUC = 0.970; best diagnostic marker [13]
miR-10a-5p	CSF exosomes (47 PD, 28 AD, 27 controls)	↑ Upregulated	Overexpressed in PD; linked to cholinergic synapse	AUC = 0.900; sensitivity 95%, specificity 95% [13]
let-7g-3p	CSF exosomes (47 PD, 28 AD, 27 controls)	↑ Upregulated	Elevated in PD; associated with neuroinflammation	AUC = 0.950; strong PD biomarker [13]
miR-331-5p	Plasma exosomes (52 PD, 48 controls)	↑ Upregulated	Elevated in PD, a potential diagnostic marker	AUC = 0.849; sensitivity 88%, specificity 85% [27]
miR-505	Plasma exosomes (52 PD, 48 controls)	↓ Downregulated	A decrease in PD, related to synaptic dysfunction	AUC = 0.898; sensitivity 90%, specificity 87% [27]
miR-195	Serum exosomes (109 PD, 40 controls)	↑ Upregulated	Increased in PD; linked to neurodegeneration pathways	AUC = 0.697; sensitivity 82%, specificity 55% [12]
miR-19b	Serum exosomes (109 PD, 40 controls)	↓ Downregulated	Lower in PD; involved in neuronal survival	AUC = 0.753; sensitivity 69%, specificity 77% [12]
miR-24	Serum exosomes (109 PD, 40 controls)	↑ Upregulated	Elevated in PD; related to neuronal protection	AUC = 0.908; high accuracy [12]
miR-23a	Serum (30 PD, 30 controls)	↑ Upregulated	Strongly increased in PD	AUC = 0.86; sensitivity 87%, specificity 76% [45]
miR-29a	Serum (30 PD, 30 controls)	↑ Upregulated	Linked to neuroinflammation and synaptic function	AUC = 0.723; sensitivity 72%, specificity 65% [45]
let-7d	Serum (30 PD, 30 controls)	↑ Upregulated	Altered in PD and vascular parkinsonism	AUC = 0.753; sensitivity 62%, specificity 87% [45]
miR-222	Serum (30 PD, 30 controls)	↑ Upregulated	Involved in the inflammatory response in PD	AUC = 0.816; sensitivity 72%, specificity 85% [45]

Key Takeaways:

1. Exosomal miRNAs in CSF and plasma have strong diagnostic potential to distinguish Parkinson’s disease from healthy controls and Alzheimer’s disease.
2. miR-1, miR-153 and miR-195 act directly on SNCA. The downregulation of these proteins in PD serum and exosomes increases α-synuclein levels, promoting the formation of Lewy bodies, a hallmark of PD.
3. miR-19b-3p and miR-24 target LRRK2. Their downregulation in PD leads to the overexpression of LRRK2, which activates the NF-κB and NLRP3 inflammasome signaling pathways, contributing to neuroinflammation.
4. miR-23a, miR-409-3p, and miR-505 act on PGC-1α and NRF2. Dysregulation (e.g., upregulation of miR-409-3p) in PD impairs mitochondrial biogenesis and antioxidant defense, increasing ROS and neuronal damage.
5. miR-10a-5p, let-7g-3p, and let-7d act on BCL2 and IGF1. Altered levels in Parkinson’s disease promote apoptosis and decrease neurotrophic support, accelerating the loss of dopaminergic neurons.

Some circulating miRNAs distinguish PD not only from healthy aging but also from vascular parkinsonism, a disease with overlapping motor features. miR-23a, miR-29a, let-7d, and miR-222 have different expression profiles, with miR-23a showing the strongest diagnostic performance (AUC = 0.86) [45]. Notably, miR-222 is associated with neuroinflammatory signaling pathways, highlighting the role of inflammation in the progression of PD.

These results support the potential of exosomal miRNAs as disease-specific biomarkers with diagnostic and prognostic utility. However, as we have previously discussed, translating these findings into clinical practice requires standardizing exosome isolation protocols, conducting large-scale validation studies, and optimizing detection methods to ensure reproducibility and clinical applicability. Future research should also investigate whether these miRNAs reflect disease progression or response to treatment, paving the way for biomarker-guided precision medicine in PD.

## 6. Integrative Hypothesis Involving miRNAs in Parkinson’s Disease

### 6.1. Based on the Target Molecules Regulated by miRNAs in Parkinson’s Disease

In PD, neuroinflammation plays a crucial role in disease progression, and an imbalance between proinflammatory and anti-inflammatory signaling pathways may exacerbate neuronal loss. Proinflammatory pathways are driven by LRRK2 mutations that amplify NF-κB signaling via MEKK3/NF-κB and MAVS, leading to increased TNF-α and IL-1β production through NLRP3 inflammasome activation (enhanced by NFAT5/NLRP3 under cellular stress), while GSK-3β further promotes NF-κB activity and STAT3 upregulates proinflammatory cytokines such as IL-6. At the same time, calpain 1/CDK5 and IGF1 contribute to neuroinflammation by activating glial cells, and LASP1 enhances immune cell infiltration. On the anti-inflammatory side, FoxO1 and TET2 promote autophagy (along with P62/p38 autophagy) by eliminating α-synuclein aggregates and decreasing NLRP3 activation. At the same time, SOCS1/Nrf2 and FSTL1 suppress JAK/STAT and NF-κB signaling, limit AKT signaling (via mTOR), and regulate excessive Bim-mediated immune responses by inducing apoptosis of overactive immune cells. However, in PD, this balance tips toward inflammation, as ROCK1/ROCK2-mediated cytoskeletal changes impair microglial clearance of α-synuclein (STAT3/α-synuclein interaction), leading to reduced anti-inflammatory signaling (e.g., via extensive transcriptional signaling). For example, via its extensive transcriptional role, SP1 is unable to counteract the proinflammatory cascade, leading to persistent neuroinflammation and loss of dopaminergic neurons. This hypothesis suggests that targeting the molecular network by strengthening FoxO1, TET2, and SOCS1 while inhibiting LRRK2, GSK-3β, and NLRP3 could restore the inflammatory balance and slow disease progression.

### 6.2. Based on miRNA Dysregulation, Verified Gene Targets, and Functional Impact in Parkinson’s Disease

In Parkinson’s disease, a dysregulated network of miRNAs orchestrates the progression of neurodegeneration by targeting genes involved in α-synuclein accumulation, neuroinflammation, mitochondrial dysfunction, and oxidative stress, ultimately leading to the loss of dopaminergic neurons. Specifically, the downregulation of miR-7, miR-124, miR-195, miR-29a/c, miR-218-5p, and miR-221-3p does not suppress the expression of α-synuclein (SNCA) and LRRK2, whereas the upregulation of miR-155-5p, miR-135b, and miR-20a-5p exacerbates inflammation by targeting SOCS1 and enhancing NLRP3 inflammasome activity. At the same time, miR-93, miR-335, miR-150, and miR-190 impair mitochondrial function and increase oxidative stress by targeting genes such as PGC-1α and NRF2. miR-30e, miR-185, miR-375, and let-7a disrupt neuronal survival pathways by targeting IGF1 and BCL2, leading to an overall loss of dopaminergic neurons in the substantia nigra.

In Parkinson’s disease, downregulation of protective miRNAs (miR-7, miR-124, miR-195, miR-29a/c, miR-218-5p, miR-221-3p, and miR-101a-3p) allows for uncontrolled accumulation of α-synuclein and LRRK2, thereby activating the NF-κB and NLRP3 inflammasome signaling pathways, which are further enhanced by the upregulation of miR-155-5p, miR-135b, and miR-20a-5p via the suppression of SOCS1. This proinflammatory milieu, in conjunction with mitochondrial dysfunction triggered by miR-93, miR-335, miR-150, and miR-190, increases oxidative stress and damages dopaminergic neurons. At the same time, the dysregulation of miR-30e, miR-185, miR-375, and let-7a promotes apoptosis, leading to the progressive loss of dopaminergic neurons in the substantia nigra, a hallmark of Parkinson’s disease. This hypothesis integrates most of the investigated miRNAs into a coherent framework, linking their verified gene targets to the major pathological processes of Parkinson’s disease. It suggests that interfering with this miRNA network may offer therapeutic potential for attenuating the progression of Parkinson’s disease.

### 6.3. Based on the Target Molecules Regulated by miRNAs in Serum Exosomes in Parkinson’s Disease

The downregulation of miR-1, miR-153, and miR-195, which are frequently observed in PD serum, cannot suppress the expression of α-synuclein (SNCA), as these miRNAs directly target SNCA mRNA, leading to increased α-synuclein aggregation and subsequent microglia activation. In addition, miR-19b-3p and miR-24, which are frequently downregulated in PD, target LRRK2 and promote its overexpression, further enhancing NF-κB-mediated neuroinflammation through the production of proinflammatory cytokines such as TNF-α and IL-1β. miR-29a and miR-222, which are also frequently altered in PD, target anti-inflammatory signaling pathways (e.g., miR-29a targets NLRP3 and miR-222 targets SOCS1), and their dysregulation (downregulation of miR-29a and upregulation of miR-222) enhances NLRP3 inflammasome activity, which exacerbates neuroinflammation. Meanwhile, mitochondrial dysfunction and oxidative stress are driven by miR-23a, miR-409-3p, and miR-505, which target PGC-1α and NRF2, key regulators of mitochondrial biogenesis and antioxidant defense. Their altered expression (e.g., upregulation of miR-409-3p and miR-505) impairs mitochondrial function and increases reactive oxygen species (ROS), which damage neurons. In addition, miR-10a-5p and let-7g-3p, which are frequently dysregulated in PD exosomes, target BCL2, an anti-apoptotic gene; their altered levels (e.g., upregulation) promote neuronal apoptosis. Conversely, let-7d and miR-331-5p reduce neurotrophic support by IGF1, further contributing to the loss of dopaminergic neurons. This interconnected miRNA network, detectable in the serum and exosomes of PD patients, reflects a systemic imbalance that drives PD pathology by promoting α-synuclein aggregation, perpetuating neuroinflammation, impairing mitochondrial function, and accelerating neuronal death. This hypothesis unifies the majority of the studied miRNAs into a cohesive framework, connecting their confirmed gene targets to key pathological processes in Parkinson’s disease, and proposes that targeting this miRNA network could provide therapeutic opportunities to slow disease progression.

### 6.4. Integrative Interactome Analysis of Exosomal miRNA Networks in Parkinson’s Disease

**Figure 3:**
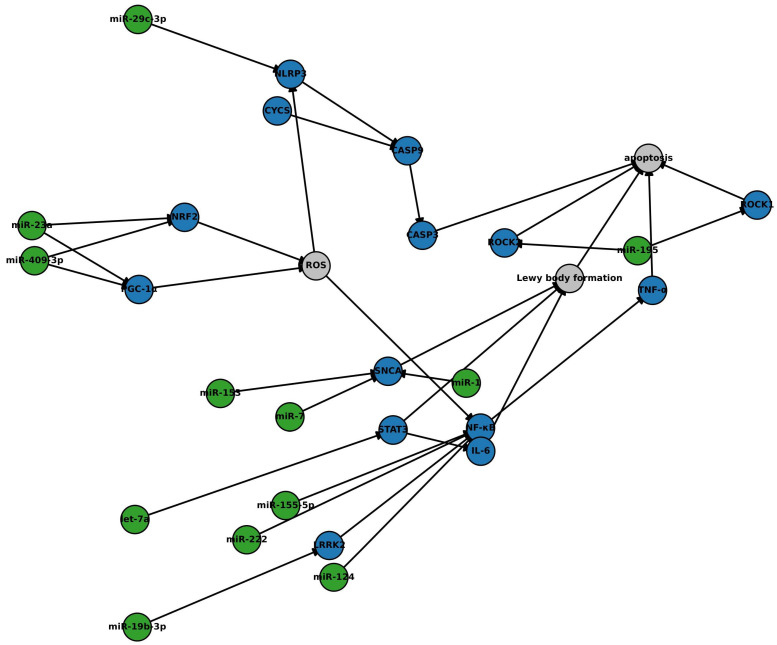
Network of microRNA–mRNA interactions implicated in Parkinson’s disease. This original figure illustrates an integrative network of exosomal microRNAs (miRNAs) and their regulatory influence on key pathogenic mechanisms in Parkinson’s disease (PD). The network incorporates high-confidence protein–protein interactions (blue nodes), miRNA–target relationships (green nodes), and neurodegenerative processes (grey nodes), including oxidative stress, neuroinflammation, mitochondrial dysfunction, and apoptosis. Exosomal miRNAs modulate critical molecular hubs, such as SNCA, NF-κB, NLRP3, PGC-1α, and NRF2, that contribute to dopaminergic neuronal vulnerability. Downregulation of neuroprotective miRNAs (e.g., miR-7, miR-124, miR-195, and miR-29c-3p) and upregulation of proinflammatory miRNAs (e.g., miR-155-5p and miR-222) exacerbate α-synuclein accumulation, inflammasome activation, and caspase-mediated neuronal death. Interaction data were curated from miRTarBase and TargetScan for miRNA–mRNA links and STRING v11.5 for protein–protein interactions. Network construction and visualization were performed using Cytoscape version 3.9.1. This model highlights candidate regulatory nodes of high translational relevance for biomarker development and therapeutic targeting in PD. Abbreviations: SNCA, α-synuclein; LRRK2, leucine-rich repeat kinase 2; NF-κB, nuclear factor kappa B; NLRP3, NOD-, LRR-, and pyrin domain-containing protein 3; CASP9, caspase 9; CASP3, caspase 3; ROCK1, Rho-associated protein kinase 1; ROCK2, Rho-associated protein kinase 2; STAT3, signal transducer and activator of transcription 3; IL-6, interleukin-6; TNF, tumor necrosis factor; PGC-1α, peroxisome proliferator-activated receptor gamma coactivator 1-alpha; NRF2, nuclear factor erythroid 2-related factor 2; CYCS, cytochrome c; ROS, reactive oxygen species.

Exosomal miRNAs regulate critical molecular pathways associated with Parkinson’s disease (PD), including α-synuclein aggregation, mitochondrial dysfunction, oxidative stress, neuroinflammation, and apoptotic signaling. To provide a systems-level perspective on how these regulatory mechanisms intersect, we developed an enhanced interactome model that integrates validated miRNA–target gene interactions with protein–protein interaction (PPI) networks relevant to PD pathology (Figure 3).

Construction of the model involved three steps. First, experimentally validated miRNA–mRNA target interactions were curated from miRTarBase (Release 9.0) and TargetScanHuman (v8.0). Only high-confidence interactions with functional validation (e.g., luciferase assays and Western blot) were included. Second, protein–protein interactions among target gene products were obtained from STRING (v11.5), filtered for interactions with a minimum confidence score of 0.7 (high confidence). Third, the composite network was visualized and analyzed using Cytoscape (v3.9.1). Topological features such as degree centrality and cluster coefficients were assessed to identify key regulatory hubs and convergence points.

The resulting network highlights how downregulation of neuroprotective miRNAs (e.g., miR-7, miR-124, miR-195, miR-153, miR-29c-3p, miR-19b-3p, miR-23a, and miR-409-3p) and upregulation of proinflammatory miRNAs (e.g., miR-155-5p and miR-222) converge on central molecular nodes, including SNCA, NF-κB, NLRP3, PGC-1α, and NRF2. These hubs integrate inflammatory, oxidative, and apoptotic pathways, which are exacerbated when regulatory control by miRNAs is lost.

Specifically, miR-7, miR-153, and miR-1 typically suppress SNCA expression, mitigating α-synuclein accumulation. miR-124, miR-195, miR-29c-3p, and let-7a regulate components of the NF-κB and NLRP3 inflammasome axis. Meanwhile, miR-409-3p and miR-23a maintain mitochondrial resilience by targeting PGC-1α and NRF2, which are critical for antioxidant defense. Dysregulation of these miRNAs drives a feedforward loop of reactive oxygen species (ROS) accumulation, inflammasome activation, and caspase-mediated apoptosis.

This integrative model provides a mechanistic framework for understanding how miRNA dysregulation contributes to PD progression, offering a basis for prioritizing candidate miRNAs for biomarker development and therapeutic intervention. Future studies should incorporate dynamic, time-resolved datasets from longitudinal patient cohorts and validate these regulatory relationships in disease-relevant experimental systems, such as iPSC-derived dopaminergic neurons or 3D brain organoids.

## 7. Clinical Applicability and Therapeutic Potential of Exosomal miRNAs in Neurodegenerative Processes

**Figure 4:**
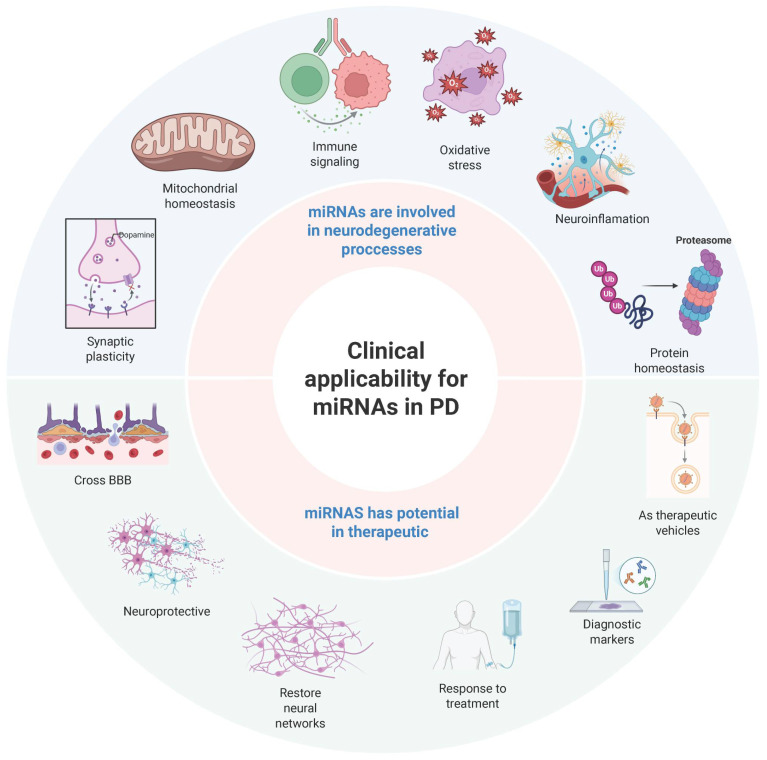
Mechanistic and clinical roles of microRNAs in Parkinson’s disease. Schematic representation of the dual role of microRNAs in Parkinson’s disease, integrating both their mechanistic involvement and clinical applicability. The figure illustrates key neurodegenerative processes regulated by microRNAs, including mitochondrial homeostasis, immune signaling, oxidative stress, neuroinflammation, protein homeostasis, and synaptic plasticity. It also highlights the therapeutic potential of microRNAs, including their ability to cross the blood–brain barrier (BBB), restore neural networks, and modulate disease progression. Additionally, the figure highlights their emerging role as diagnostic biomarkers and vehicles for targeted therapeutic delivery. This conceptual framework synthesizes current evidence on the pathogenic and translational significance of microRNAs in Parkinson’s disease. Created with BioRender.com (BioRender 3.5).

miRNAs regulate gene expression in numerous biological systems, including the central nervous system (CNS) [8]. Their roles in synaptic plasticity, mitochondrial homeostasis, and immune signaling are essential for maintaining neuronal function. In PD, dysregulated miRNA signaling pathways disrupt several processes, including the response to oxidative stress, neuroinflammation, and protein homeostasis, contributing to neurodegeneration [46] (Figure 4).

miRNA biogenesis takes place in a strictly regulated process. Primary miRNAs (pri-miRNAs) are transcribed and processed by Drosha and its cofactor DGCR8, resulting in precursor miRNAs (pre-miRNAs) that are transported into the cytoplasm via exportin-5. In the cytoplasm, Dicer processes the pre-miRNAs into mature miRNAs, which are incorporated into the RNA-induced silencing complex (RISC) to regulate gene expression by targeting specific mRNAs for degradation or translational repression [8]. Advances in high-throughput sequencing and cryo-electron microscopy (cryo-EM) have identified key sequence motifs that control miRNA processing, highlighting their specificity and functional complexity [8]. While most miRNAs follow canonical processing pathways, alternative mechanisms, including mirtron-derived miRNAs and Argonaute-dependent maturation, extend their regulatory capabilities.

Exosomal miRNAs have gained recognition as key mediators of intercellular communication and potential biomarkers in PD. Exosomes are 30–150 nm extracellular vesicles secreted by neurons and glia, facilitating the transfer of miRNAs, proteins, and lipids, and enabling long-range molecular signaling within the brain [13]. In contrast to free miRNAs, exosomal miRNAs are encapsulated in vesicles that protect them from degradation and enable them to regulate mitochondrial function, neuroinflammation, and oxidative stress, which play central roles in the pathogenesis of PD [46].

There is growing evidence that exosomal miRNAs contribute to disease progression. miR-34a, for example, increases oxidative stress and neuroinflammation, and promotes the loss of dopaminergic neurons [12]. Similarly, let-7a enhances α-synuclein aggregation and activates Toll-like receptor 7 (TLR7), thereby enhancing neuroinflammatory responses [12]. Conversely, neuroprotective exosomal miRNAs can attenuate PD pathology. miR-137 increases cellular resistance to oxidative stress [46], while miR-7116-5p reduces TNF-α-driven neuroinflammation [15]. Importantly, circulating exosomal miRNAs in CSF and blood correlate with disease severity, highlighting their potential as non-invasive biomarkers [14,27].

Exosomal miRNAs show promise as novel therapeutics for PD. Their ability to cross the BBB makes them attractive candidates for targeted miRNA-based interventions. Engineered exosomes carrying neuroprotective microRNAs (miRNAs) can modulate molecular signaling pathways associated with PD with high specificity and minimal off-target effects [13]. Preclinical studies suggest that modulating exosomal miRNA content could help restore disrupted molecular networks, potentially slowing or reversing the progression of PD [46] (Figure 4, lower part). However, significant barriers to clinical translation remain.

As we have already mentioned, the current methods have various advantages and limitations in terms of specificity, yield, and scalability. One major limitation is the isolation and purification of exosomes. This has already been discussed in the first part of this review. Another challenge is differentiating true exosomal miRNAs from non-vesicular extracellular miRNAs, as a significant proportion of circulating miRNAs are associated with protein complexes or lipoproteins, rather than exosomes [13]. Understanding miRNA sorting in exosomes and their selective uptake by recipient neurons is critical for optimizing diagnostic and therapeutic applications [14]. The clinical implementation of miRNA-based therapies also requires overcoming issues related to exosome stability, therapeutic scalability, and regulatory approval before these interventions can be introduced into routine clinical practice [13]. The lack of standardized protocols complicates the validation of biomarkers, leading to varying study results [46].

## 8. Exosomal miRNAs in Parkinson’s Disease: From Bench to the Bedside

miRNAs are crucial regulators of gene expression, playing a significant role in neuronal survival, synaptic plasticity, and neuroinflammatory processes. In recent years, they have emerged as promising candidates for therapeutic and diagnostic applications in PD, as they can modulate several cellular processes. However, translating miRNA-based strategies into clinical practice is associated with significant challenges, particularly in the targeted delivery of miRNA to the brain and the standardization of biomarkers.

### 8.1. Exosomal miRNAs as Therapeutic Vehicles

Exosomes, small extracellular vesicles involved in intercellular communication, have garnered attention as a natural delivery system for miRNAs. Unlike synthetic carriers or viral vectors, exosomes can cross the BBB, enabling targeted therapeutic delivery to neurons affected by PD [13]. Their biocompatibility and ability to deliver disease-modifying miRNAs make them a promising platform for neuroprotective interventions (Figure 4, bottom part).

Preclinical studies have demonstrated the potential of exosome-loaded miRNAs in PD models. miR-7 and miR-124, when administered via exosomes, suppress neuroinflammation, reduce oxidative stress, and protect dopaminergic neurons from degeneration [14,27,47]. Similarly, miR-195 and miR-330, which inhibit inflammatory cascades, have been shown to counteract microglial activation and prevent neuronal apoptosis [28]. These results suggest that exosome-mediated miRNA therapy may represent a targeted and minimally invasive strategy for neuroprotection in PD.

### 8.2. Exosomal miRNAs and Disease Progression

Apart from their therapeutic potential, exosomal miRNAs play a fundamental role in neuronal communication and repair. Neurons, astrocytes, and microglia exchange molecular signals via exosomes, which are crucial for regulating neuroinflammation and coordinating cellular responses to injury [12]. In PD, this communication network is increasingly disrupted, contributing to neuronal loss and neuroinflammation. For example, miR-34a-enriched exosomes from astrocytes have been shown to increase oxidative stress and trigger microglial activation, which further accelerates neurodegeneration [13]. Strategies to restore protective miRNAs or inhibit pathogenic miRNAs could open up new therapeutic opportunities. However, deciphering the exact function of individual miRNAs remains a challenge, as their effects are often context-dependent and influenced by the stage of the disease.

### 8.3. Exosomal miRNAs as Diagnostic Biomarkers

The search for non-invasive, reliable biomarkers for PD remains a priority, as early diagnosis is crucial for timely intervention and treatment. Circulating exosomal miRNAs in CSF and plasma have demonstrated their diagnostic potential, providing insight into the pathophysiology of the disease before clinical symptoms appear (Figure 4, bottom part). Several studies have shown that miR-153 and let-7g-3p are elevated in CSF, while miR-1 and miR-19b-3p are significantly reduced in patients with PD [13]. In addition, exosomal miR-331-5p and miR-505, found in plasma, have demonstrated high diagnostic specificity, with area under the curve (AUC) values above 0.85 [27]. In contrast to conventional biomarkers that rely on late clinical manifestations, exosomal-miRNA-based diagnostics could facilitate early detection of the disease and improve monitoring of disease progression and therapeutic response.

However, the clinical translation of exosomal miRNA biomarkers faces several obstacles. Variability in miRNA expression across individuals, the impact of comorbidities, and inconsistencies in laboratory protocols complicate efforts to standardize [46]. Developing high-throughput, reproducible detection methods will be critical for ensuring clinical applicability.

### 8.4. Translational Challenges and Future Directions for Exosomal miRNAs in PD

Despite their therapeutic promise, miRNA-based diagnostics and interventions in PD face considerable challenges. One of the primary obstacles lies in the intrinsic complexity of miRNA regulatory networks. Individual miRNAs frequently target multiple genes and influence diverse signaling pathways, which raises concerns about off-target effects and unintended biological consequences. Achieving therapeutic specificity while preserving physiological regulatory balance remains a critical goal for clinical translation.

Exosome-based delivery systems offer a biologically relevant platform for miRNA therapies, owing to their ability to cross the blood–brain barrier and facilitate intercellular communication. However, the clinical application of these systems requires further optimization. Challenges, such as low scalability, variable reproducibility, and limited exosome stability, under clinical conditions hinder the achievement of consistent therapeutic outcomes. The absence of standardized protocols for exosome isolation, miRNA cargo loading, and targeted delivery impairs therapeutic efficacy and may provoke immune responses if not properly addressed [13].

Another unresolved issue concerns the specificity of exosomal miRNAs as biomarkers for PD. Several miRNAs associated with PD—such as miR-29a, miR-222, and let-7d—have also been reported in Alzheimer’s disease and multiple system atrophy [45]. This biological overlap complicates diagnostic discrimination and underscores the need for composite biomarker panels. Such panels could integrate multiple miRNA signatures with other molecular markers, including proteins and long non-coding RNAs, to enhance specificity and diagnostic accuracy.

To translate the potential of exosomal miRNAs into clinical tools, several research directions must be pursued. First, advancements in exosome engineering are required to improve the stability, targeting specificity, and therapeutic efficacy of miRNA cargo. Second, the development of standardized, high-throughput protocols for miRNA detection and quantification is essential to ensure reproducibility and diagnostic reliability. Third, further mechanistic studies are needed to elucidate the processes that govern the sorting of miRNA into exosomes and their uptake by recipient neurons or glial cells. Understanding these molecular pathways will be critical for designing precise and efficient therapeutic delivery strategies. Finally, large-scale clinical trials are necessary to evaluate the long-term safety, efficacy, and feasibility of miRNA-based interventions in diverse PD populations.

The application of exosomal miRNAs in PD diagnosis and therapy represents a conceptual shift from symptomatic management to molecularly targeted, precision-based approaches. Although promising, the field must overcome substantial biological and technological hurdles before these strategies can be implemented in routine clinical care. Continued progress in RNA biology, nanomedicine, and biomarker discovery will be instrumental in transforming exosomal miRNA research into viable tools for early diagnosis, disease monitoring, and disease-modifying therapies in PD.

## 9. Future Perspectives

This review highlights the translational potential of exosomal microRNAs (miRNAs) in Parkinson’s disease (PD), particularly in modulating oxidative stress, neuroinflammation, and mitochondrial dysfunction—key contributors to dopaminergic degeneration. The ability of exosomal miRNAs to cross the blood–brain barrier (BBB) positions them as promising candidates for the development of diagnostic biomarkers and therapeutic agents. However, several scientific and technical challenges must be addressed before these insights can be translated into clinical practice.

One of the most pressing obstacles is the efficient and targeted delivery of therapeutic miRNAs across the BBB. Although exosomes provide a biologically compatible delivery system, achieving high payload concentrations in specific cell populations—such as dopaminergic neurons and activated microglia—remains suboptimal. Current isolation methods, including ultracentrifugation, size-exclusion chromatography, and immunoaffinity capture, suffer from variability in yield, purity, and scalability [13,18,46]. This lack of standardization contributes to inter-study inconsistencies and hinders the reproducibility essential for clinical validation. The development of engineered exosomes with surface ligands tailored to target specific cell types may enhance delivery specificity and therapeutic efficacy [13].

Beyond delivery, miRNA stability in circulation is a significant barrier. Endogenous and exogenous miRNAs are susceptible to degradation by extracellular RNases and rapid clearance from the bloodstream, significantly limiting their half-life and bioavailability [13,46]. Chemical modifications to the miRNA backbone, encapsulation in nanoparticles or liposomes, and exosome surface engineering are currently being explored to address these limitations. These strategies aim to maximize therapeutic stability while minimizing off-target effects and immune activation [13,46].

Another layer of complexity arises from the pleiotropic nature of miRNAs. Each miRNA can regulate multiple signaling pathways simultaneously, and its effects can vary depending on disease stage and cellular context. For instance, miR-7, miR-30e, and miR-124 have demonstrated neuroprotective effects by suppressing neuroinflammatory pathways [23,26,27], whereas miR-195, miR-155-5p, and miR-29c-3p may enhance microglial activation and promote neuroinflammation [11,24,28]. This duality highlights the importance of disease-stage-specific profiling and the application of high-throughput screening and systems biology to predict and manage downstream effects. Emerging tools, such as CRISPR-based modulation of miRNA activity and artificial intelligence-driven predictive modeling, may support these efforts [22,46].

In the diagnostic domain, exosomal miRNAs in plasma and cerebrospinal fluid (CSF) continue to demonstrate high diagnostic accuracy in distinguishing PD from other neurodegenerative conditions. Notably, the upregulation of miR-153 and let-7g-3p, along with the downregulation of miR-1 and miR-19b-3p, shows strong correlations with disease severity and progression [13,27,45]. Nevertheless, significant inter-individual variability and the absence of standardized detection and normalization protocols limit the current clinical applicability of these methods. Advancing this field will require the implementation of validated, reproducible workflows that utilize technologies, such as next-generation sequencing, quantitative PCR, and digital PCR, combined with robust normalization strategies [13,14,46].

The long-term safety, efficacy, and regulatory approval of exosome-based therapies remain underexplored. While preclinical studies support the therapeutic potential of miRNA modulation in reducing oxidative stress and protein aggregation [11,24,26], robust validation in translational models is urgently needed. The use of induced pluripotent stem cell (iPSC)-derived dopaminergic neurons, brain organoids, and advanced animal models may help evaluate dosing, delivery kinetics, immune tolerance, and adverse effects in a controlled and clinically relevant manner [13,46].

Significantly, the relevance of exosomal miRNAs extends beyond PD. Similar mechanisms involving mitochondrial dysfunction, neuroinflammation, and protein aggregation have been implicated in Alzheimer’s disease, amyotrophic lateral sclerosis, and multiple system atrophy. Comparative studies may help identify convergent miRNA targets across neurodegenerative diseases, broadening the scope of miRNA-based interventions [14,27].

Ultimately, advancing the clinical utility of exosomal miRNAs in PD will require a multidisciplinary approach that bridges neuroscience, molecular biology, bioengineering, and computational science. Refinement of exosome engineering, enhancement of miRNA stability and delivery, and standardization of biomarker detection pipelines are key priorities for the field. The integration of nanotechnology, CRISPR-based editing [22], and AI-driven miRNA profiling is poised to transform the field.

By framing exosomal miRNA research within these strategic and technical challenges, this discussion outlines a roadmap for its clinical translation. Exosomal miRNAs hold the potential to redefine the diagnosis, monitoring, and treatment of PD. Addressing the barriers identified here will be essential to unlocking their full therapeutic promise and delivering personalized, disease-modifying interventions for patients with Parkinson’s disease and related neurodegenerative disorders.

## Data Availability

No new data were created or analyzed in this study. The analysis in our manuscript is based entirely on the data summarized in the tables. We did not perform a meta-analysis or any additional statistical modeling.

## Abbreviations

The following abbreviations are used in this manuscript:

AUC	Area under the curve
BBB	Blood–brain barrier
CSF	Cerebrospinal fluid
EVs	Extracellular vesicles
miRNA	MicroRNA
MPP+	1-Methyl-4-phenylpyridinium
NGS	Next-generation sequencing
PD	Parkinson’s disease
qPCR	Quantitative polymerase chain reaction
ROS	Reactive oxygen species
